# Detection of pandemic influenza A/H1N1/pdm09 virus among pigs but not in humans in slaughterhouses in Kenya, 2013–2014

**DOI:** 10.1186/s13104-019-4667-4

**Published:** 2019-09-24

**Authors:** Eric Mogaka Osoro, Shirley Lidechi, Jeremiah Nyaundi, Doris Marwanga, Athman Mwatondo, Mathew Muturi, Zipporah Ng’ang’a, Kariuki Njenga

**Affiliations:** 1 Jomo Kenyatta University of Agriculture and Technology, Nairobi, Kenya; 2 Kenya Medical Research Institute, Nairobi, Kenya; 3 Ministry of Health, Nairobi, Kenya; 4 Ministry of Agriculture and Irrigation, Nairobi, Kenya; 5 Washington State University, Pullman, USA

**Keywords:** Swine, Human, Influenza A virus, Surveillance

## Abstract

**Objective:**

We conducted four cross-sectional studies over 1 year among humans and pigs in three slaughterhouses in Central and Western Kenya (> 350 km apart) to determine infection and exposure to influenza A viruses. Nasopharyngeal (NP) and oropharyngeal (OP) swabs were collected from participants who reported acute respiratory illness (ARI) defined as fever, cough or running nose. Nasal swabs and blood samples were collected from pigs. Human NP/OP and pig nasal swabs were tested for influenza A virus by real-time reverse transcriptase polymerase chain reaction (PCR) and pig serum was tested for anti-influenza A antibodies by ELISA.

**Results:**

A total of 288 participants were sampled, 91.3% of them being male. Fifteen (5.2%) participants had ARI but the nine swabs collected from them were negative for influenza A virus by PCR. Of the 1128 pigs sampled, five (0.4%) nasal swabs tested positive for influenza A/H1N1/pdm09 by PCR whereas 214 of 1082 (19.8%) serum samples tested for Influenza A virus antibodies. There was higher seroprevalence in colder months and among pigs reared as free-range. These findings indicate circulation of influenza A/H1N1/pdm09 among pigs perhaps associated with good adaptation of the virus to the pig population after initial transmission from humans to pigs.

## Introduction

Influenza A viruses circulate widely in animals, including birds, humans, pigs, and other mammals, and frequently cause severe epidemics and pandemics that affect both animals and humans [1–4]. The most recent influenza pandemic was in 2009, which was caused by a novel pig-origin influenza A virus resulting in > 500,000 human deaths globally [5]. A common mechanism of emergence of novel influenza viruses is acquisition of new antigenic material during an inter-species transmission event [3, 6].

Pigs, long believed to be a mixing vessel for inter-species influenza virus transmission, can be a source of swine influenza infection to humans occupationally exposed to them [7]. Pig slaughterhouses present a particularly prime environment for pig-to-human transmission of influenza A viruses, and with increasing pig farming in low biosafety and biosecurity settings in Kenya, the level of human exposures to swine influenza viruses has increased [8]. Few studies on influenza virus transmission at the human-animal interface have been conducted in sub-Saharan Africa, including one in a Kenyan pig slaughterhouse that detected A/H1N1/pdm09 among pigs, suggesting introduction from humans [9].

To mitigate the severity of influenza pandemics, early detection through syndromic surveillance in humans is key [3]. Even though human influenza surveillance in Kenya has improved by targeting acute respiratory illness at sentinel sites, there is no emphasis on people occupationally exposed to pigs or birds. Here, we conducted a series of cross-sectional studies among human and pigs in three pig slaughterhouses to determine infection and exposure to influenza A viruses.

## Main text

### Methods

**Fig. 1:**
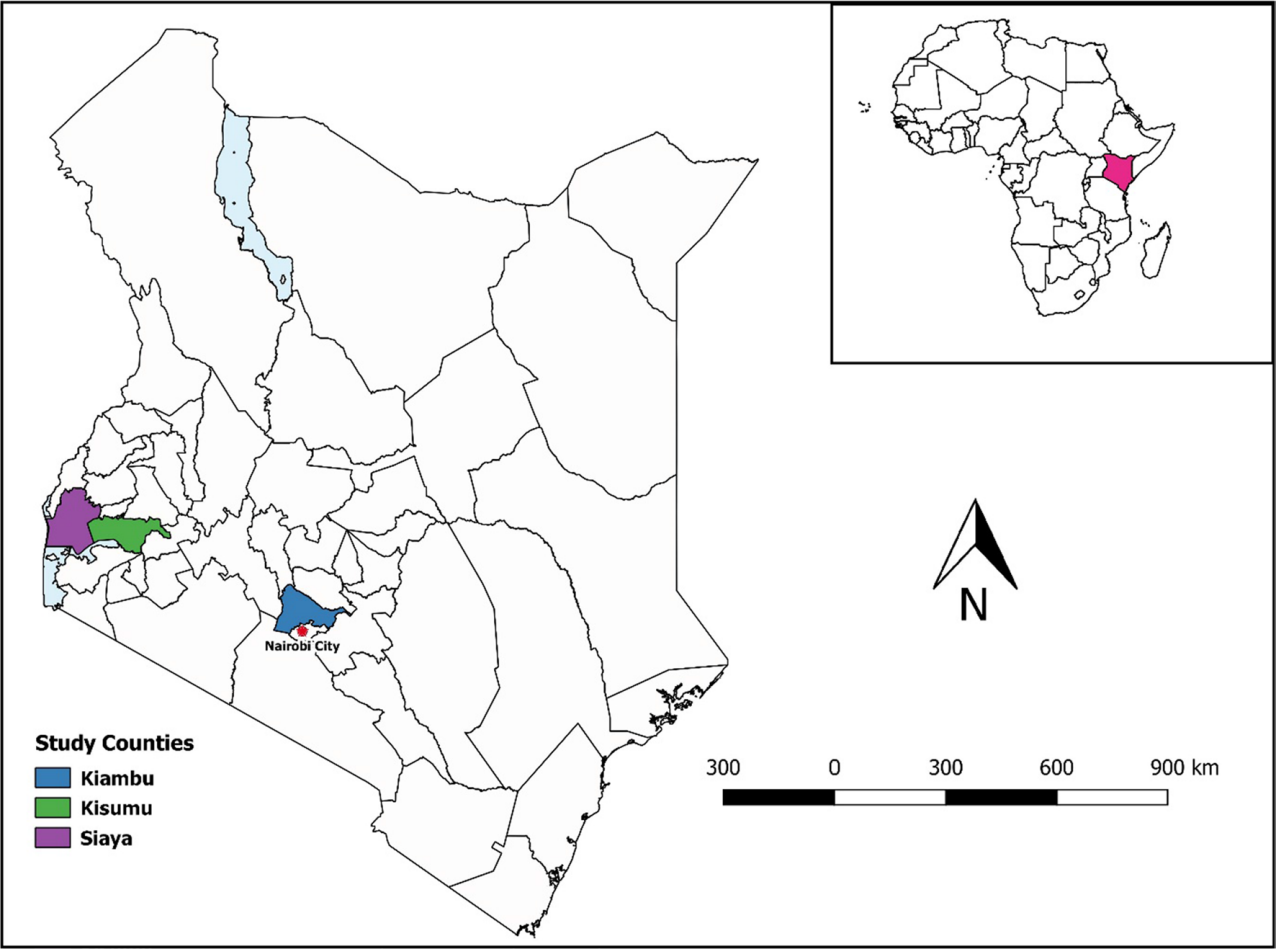
Map of Kenya showing the three counties where the sampling was conducted. Inset is a map of Africa with Kenya highlighted. Map created in QGIS

We conducted four cross-sectional studies over a period of 1 year among humans and pigs in three slaughterhouses in Kiambu (Uthiru slaughterhouse), Kisumu and Siaya (Bondo slaughterhouse) counties (Fig. 1). Kiambu County in central Kenya features farms that have intensive pig production system while farms in Kisumu and Siaya counties in Western Kenya employ extensive pig production systems. These contrasting pig production systems represent varying degrees of contact between humans and pigs.

### Sample size and sampling

All pig slaughterhouse workers, farmers and traders who visited the slaughterhouses to deliver pigs or to purchase meat, animal health personnel and others working in the slaughterhouses were requested to participate in the study. For pig sampling, an estimated seroprevalence of 20% was assumed, precision level of 5% and at 95% confidence level, giving a minimum sample size of 246 per sampling period. The slaughterhouses were visited each consecutive working day for 10 days to sample the pigs. For Uthiru (Kiambu) slaughterhouse, we sampled every other pig to a maximum of 25 per day. For Kisumu and Bondo (Siaya) slaughterhouse where volume of pigs slaughtered is low, all the pigs presented for slaughter on each day of sampling were targeted for sampling.

### Human and animal sample collection

Nasopharyngeal (NP) and Oropharyngeal (OP) swabs were collected from participants reporting acute respiratory illness during sampling. Acute respiratory illness (ARI) was defined as reported cough, runny nose or sore throat. The swabs were placed in cryovials with virus transport medium (VTM) and shipped to Kenya Medical Research Institute (KEMRI) laboratories in Nairobi on ice, where they were preserved at − 80 °C until testing.

Nasal swabs and blood samples were collected from pigs; nasal swabs were placed in cryovials with VTM and shipped to KEMRI laboratories in Kisumu for storage at − 80 °C until testing. Blood samples were processed for sera on the same day of collection and stored at − 80 °C until testing. All testing of animal samples was at the KEMRI laboratories.

### Serological and molecular testing for influenza A virus

We used IDEXX^®^ ELISA kit (FlockChek AI MultiS-Screen Ab Test Kit^®^, Westbrook, Maine) to test animal serum for influenza A virus antibodies, following manufacturer instructions. We applied an adjusted cut-off of the S/N of < 0.673 for pig sera, which had been shown to increase sensitivity and specificity [10].

We used real-time reverse transcriptase polymerase chain reaction (RT-PCR) to test human NP/OP samples for influenza A virus RNA by applying primers and probes against the matrix gene of influenza A and NS1 gene of influenza B viruses [11]. A cycle threshold (C_T_) value of ≤ 40 was the cut-off for positivity. Positive and negative controls were used to validate test assay.

Pig nasal swabs were tested for influenza A virus by RT-PCR using the CDC protocol for influenza A virus detection [12]. The Influenza A sub-typing utilized oligonucleotides targeting hemagglutinin and neuraminidase genes (contemporary human pandemic H1, human AH3, AH7, AH5, N1 and N2) of swine influenza viruses [13].

### Data collection and analysis

A structured questionnaire was administered to participants to collect data on sociodemographic characteristics, frequency and level of contact with pigs, influenza vaccination history and acute respiratory illness using Personal Digital Assistant devices running on a windows-based application. Data were cleaned and analyzed by the R statistical software [14]. Descriptive statistics were conducted for socio-demographic characteristic by pig exposure status. Pig exposure was defined as any person who routinely skinned pigs, stunned pigs, sold pork or offals at the slaughterhouse. Categorical and continuous variables were compared using Chi Square test (or Fishers exact test) and Student t-test, respectively. Prevalence was determined as proportion of samples positive for influenza A virus against all samples tested.

The study was approved by the KEMRI Scientific and Ethical Review Committee and the Animal Care and Use Committee and all participants gave written informed consent before enrolment.

### Results

All three slaughterhouses operated for 5 days a week (Monday to Friday), receiving pigs from farms within the respective and neighboring counties. The Uthiru slaughterhouse received an average of 50 pigs per day while the Bondo and Kisumu slaughterhouses received 3 to 5 pigs per day. All pigs slaughtered in the three slaughterhouses were adults.

A total of 288 participants were sampled over the four sampling periods, 91 (31.6%) in September 2013, 43 (14.9%) in December 2013, 101 (35.1%) in May 2014, and 53 (18.4%) in September 2014. More than half (51.7%) of the participants were from Uthiru slaughterhouse.

Majority of participants were male (91.3%), and 35.4% (n = 102) of them were classified as pig exposed. The mean age for the participants was 35.5 years with a significant difference between the mean age of pig exposed (32.5 years) and non-pig exposed persons (37.2 years). Although 55.9% of all participants had completed at least secondary education, 5% of non-pig exposed had no formal education whereas all pig exposed participants had some formal education (Table 1).

**Table 1:** Sociodemographic characteristics of participants by pig exposure status, 2013–2014

Characteristic	Categories	Pig exposure		Total	p-value
		Yes n (%)	No n (%)		
Sex	Female	6 (5.9)	19 (10.2)	25 (8.7)	0.212
	Male	96 (94.1)	167 (89.8)	263 (91.3)	
Age in years	Mean (SD)	32.5 (11.2)	37.2 (12.2)	35.5 (12)	0.001
Highest education level completed	No formal education	0 (0.0)	9 (4.8)	9 (3.1)	0.008
	Primary	45 (44.1)	73 (39.2)	118 (41.0)	
	Secondary	51 (50.0)	75 (40.3)	126 (43.8)	
	Post-secondary	6 (5.9)	29 (15.6)	35 (12.2)	
Occupation	Slaughterhouse worker	84 (82.4)	72 (38.7)	156 (54.2)	< 0.001
	Pig farmer	7 (6.9)	29 (15.6)	36 (12.5)	
	Pig trader	7 (6.9)	49 (26.3)	56 (19.4)	
	Other	4 (3.9)	36 (19.4)	40 (13.9)	
Sampling period	Sep, 2013	32 (31.4)	59 (31.7)	91 (31.6)	0.012
	Dec, 013	8 (7.8)	35 (18.8)	43 (14.9)	
	May, 2014	35 (34.3)	66 (35.5)	101 (35.1)	
	Sep, 2014	27 (26.5)	26 (14.0)	53 (18.4)	
Slaughterhouse	Bondo	29 (28.4)	47 (25.3)	76 (26.4)	0.210
	Kisumu	27 (26.5)	36 (19.4)	63 (21.9)	
	Uthiru	46 (45.1)	103 (55.4)	149 (51.7)	

*SD* standard deviation

Fifteen (5.2%) participants had ARI during the sampling periods. Of the 9 OP/NP swabs collected from these ARI cases, none were positive for influenza A virus.

In total, 1128 pigs were sampled (nasal swabs) for influenza testing, including 73% from Uthiru slaughterhouse. Of these, 5 pigs (0.4%) were positive for Influenza A virus RNA and all subtyped as A/H1N1/pdm09 virus. Serum was collected from 1082 pigs, 75% of them from Uthiru slaughterhouse. Of these, 214 (19.8%) pigs were positive for influenza A virus antibodies by ELISA. Samples collected in September 2014 had the highest prevalence of 37.1% (93 of 251), followed by September 2013 at 19.8% (47 of 237). Among the positive samples (n = 214), 65.4% (140) were collected in September 2013 or September 2014. Among slaughterhouses, 34.5% (30 of 87) of the samples from Bondo were seropositive, followed by 22.6% (21 of 93) in Kisumu slaughterhouse (Table 2). None of the farmers reported vaccinating their pigs against influenza.

**Table 2:** Seroprevalence of influenza A virus among pigs by sampling period and slaughterhouse, 2013–2014

	Samples tested	Positive	Seroprevalence (%)	95% CI
All samples	1082	214	19.8	17.5, 22.3
Sampling period				
Sep, 2013	237	47	19.8	15.3, 25.4
Dec, 2013	293	28	9.6	6.7, 13.5
May, 2014	301	46	15.3	11.7, 19.8
Sep, 2014	251	93	37.1	31.3, 43.2
Slaughterhouse				
Bondo	87	30	34.5	25.3, 44.9
Kisumu	93	21	22.6	15.3, 32.1
Uthiru	902	163	18.1	15.7, 20.7

*CI* confidence interval

### Discussion

We found evidence of both active influenza A virus infection and widespread exposure (seropositivity) among pigs but no infection among humans in a linked human-animal study in three slaughterhouses in Kenya. Influenza virus (A/H1N1/pdm09) virus RNA and antibodies, which is associated with seasonal human influenza in Kenya, was detected in the pig samples from central and western Kenya, suggesting either persistent human to pig transmission of influenza virus (A/H1N1/pdm09) or establishment and continued circulation of influenza virus (A/H1N1/pdm09) among pig populations [15]. This finding is consistent with a similar study in Kenya where 0.5% of sampled pigs were found to have A/H1N1/pdm09 virus [16]. Between 2016 and 2018 on average, seasonal human influenza in Kenya was associated with A/H1N1/pdm09 (32.5%), human A/H3N2 (33.8%) and influenza B (30.9%) [17].

Our study provides evidence of intense circulation of swine influenza virus among pig populations in two distinct geographical regions of Kenya, located > 350 kilometres apart, with the high average seroprevalence of 20%. The higher prevalence reported in Bondo (34.5%) and Kisumu (22.6%) slaughterhouses located in Western Kenya may be due to the free-range nature of pig production there, when compared with Uthiru slaughterhouse in the central region of the country where confined production system is practiced. Our findings also point to higher influenza transmission during the colder months (July–September) as supported by almost two-thirds of the seropositive pigs sampled during this period. In addition, all the PCR positive samples were collected during the cold season. Trends in human seasonal influenza in Kenya have also shown higher transmission during the colder months of June to August [15].

The occurrence of influenza A/H1N1/pdm09 virus in pigs has been documented in most regions of the world, including Africa where it has been reported in Kenya, Nigeria, Ghana and Cameroon [16, 18, 19]. Studies have shown that when influenza virus (A/H1N1/pdm09) circulates in local pig populations it continues to undergo antigenic changes over time [20]. The influenza A virus seroprevalence reported in our study was comparable to 17% reported in an earlier study in Kenya [16]. However, studies from other countries showed varied findings ranging from 5% in Uganda to 49% in Vietnam [21–24]. The variations in prevalence reported in the studies may be due to differences in sampling methodology (farm level vs live market vs slaughterhouses), and pig populations in the study area. The Southeast Asia region has large pig farms that likely support higher influenza virus transmission [23, 24].

In conclusion, our study reports detection of influenza virus (A/H1N1/pdm09) among pigs and high seroprevalence adding to the evidence of intense circulation among pigs from the few studies in the East Africa region. The increasing commercialization of pig farming in Kenya, provides a suitable environment for exposure and occurrence of zoonotic events related to influenza A virus [8]. The evolution of reassortant viruses and their potential transmission to humans is unpredictable making routine monitoring at the pig-human interface a priority. Virological surveillance offers a mechanism to detect early any changes in the antigenic structure or zoonotic transmission events.

## Limitations of the study

Our study had several limitations. We were not able to collect demographic data such as age, sex and farm level factors among the sampled pigs to allow for testing for associations with seropositivity. While most of the pigs brought for slaughter were mature adults, they were mostly delivered by traders who would not provide reliable farm level data on the sampled pigs. Another limitation is that we did not conduct haemagglutination inhibition (HI) tests to confirm the influenza strains circulating among pigs. However, another study in 2010–2012 [16] reported 72% of seropositive pigs had influenza virus (A/H1N1/pdm09) by HI, findings which might reflect the diversity of influenza virus among pigs in our study.

## Data Availability

The datasets used and/or analysed during the current study are available from the corresponding author on reasonable request.

## Abbreviations

ARI: acute respiratory illness
CI: confidence interval
KEMRI: Kenya Medical Research Institute
NP: nasopharyngeal
OP: oropharyngeal
PCR: real-time reverse transcriptase polymerase chain reaction
VTM: virus transport medium
